# A Study of the Oxidation Behaviour of Pile Grade A (PGA) Nuclear Graphite Using Thermogravimetric Analysis (TGA), Scanning Electron Microscopy (SEM) and X-Ray Tomography (XRT)

**DOI:** 10.1371/journal.pone.0143041

**Published:** 2015-11-17

**Authors:** Liam Payne, Peter J. Heard, Thomas B. Scott

**Affiliations:** Interface Analysis Centre, University of Bristol, Bristol, BS8 1TL, United Kingdom; VIT University, INDIA

## Abstract

Pile grade A (PGA) graphite was used as a material for moderating and reflecting neutrons in the UK’s first generation Magnox nuclear power reactors. As all but one of these reactors are now shut down there is a need to understand the residual state of the material prior to decommissioning of the cores, in particular the location and concentration of key radio-contaminants such as ^14^C. The oxidation behaviour of unirradiated PGA graphite was studied, in the temperature range 600–1050°C, in air and nitrogen using thermogravimetric analysis, scanning electron microscopy and X-ray tomography to investigate the possibility of using thermal degradation techniques to examine ^14^C distribution within irradiated material. The thermal decomposition of PGA graphite was observed to follow the three oxidation regimes historically identified by previous workers with limited, uniform oxidation at temperatures below 600°C and substantial, external oxidation at higher temperatures. This work demonstrates that the different oxidation regimes of PGA graphite could be developed into a methodology to characterise the distribution and concentration of ^14^C in irradiated graphite by thermal treatment.

## Introduction

### Background

The decommissioning of the first generation of gas-cooled, graphite-moderated Magnox reactors in the United Kingdom will lead to approximately 45,000 m^3^ of graphite waste needing disposal [1, 2]. The majority of this will be classified as intermediate level waste (ILW) [3] and includes the long lived radionuclide ^14^C, which is a key radionuclide in the assessment of the safety of a geological disposal facility (GDF) for radioactive waste [4]. This key radionuclide is formed during the lifetime of the reactor as the graphite, which is used as a moderator and reflector material, is bombarded with thermal neutrons. Neutrons may be captured by either ^13^C, ^14^N and ^17^O to form ^14^C [5], Table 1.

**Table 1:** Carbon-14 production mechanisms and cross sections, [5].

Target isotope	Mechanism	Thermal cross-section (barns)	Isotopic abundance of the parent material (%)
^14^N	^14^N (n,p) ^14^C	1.81	99.6349
^13^C	^13^C (n,*γ*) ^14^C	0.0009	1.103
^17^O	^17^O (n,*α*) ^14^C	0.235	0.0383

After final shutdown of a Magnox reactor the oxidation of graphite is not believed to be problematic for either decommissioning or disposal. This is because temperatures are not expected to be sufficient to facilitate oxidation and the criteria for self-sustained combustion are even less likely to occur [6]. However, studying the deliberate oxidation of graphite at temperatures exceeding 600°C has the potential benefit of assisting in the examination of irradiated reactor graphite samples to determine the distribution and concentration of ^14^C. This is of interest as the different possible ^14^C formation pathways may lead to variations in its distribution within the graphite, thereby altering its availability for release when emplaced in a deep geological disposal facility. The potential release of ^14^C has been previously investigated by leaching irradiated graphite in alkaline solutions [7] where it was observed that there are three potential fractions of ^14^C. The first is a labile fraction that may be released soon after disposal, composed of ^14^C on surface of the graphite. The second is a slowly releasable fraction from the near surface associated ^14^C and finally there is an essentially non-releasable fraction from ^14^C located in the graphite lattice. If the thermal oxidation behaviour of PGA graphite is known then there is the potential to selectively remove the ^14^C from the different fractions of irradiated graphite. This may offer a method for determining the labile ^14^C fraction in irradiated graphites more rapidly than leaching tests.

Lifetime monitoring of the Magnox reactor fleet, including core inspection has yielded a large inventory of small, cylindrical graphite trepan samples which have been examined and tested to assist in safety cases for extension of generation lifetime [8]. Post-mortem analysis of such samples to investigate ^14^C concentration is possible, although standard radiation measurements are limited due to ^14^C being a beta emitter and the self-shielding nature of graphite (a beta particle arising from ^14^C decay has an approximate range of only 0.013 cm in graphite [9]). Consequently, only a small percentage of the total quantity of ^14^C can be determined by direct measurements of the graphite surface. Therefore, an alternative method is required to examine the total ^14^C concentration of these samples which has historically involved the thermal degradation of the samples followed by Liquid Scintillation Counting (LSC) [10]. The thermal treatment of irradiated graphite has been studied previously [11, 12], however this aimed at achieving maximum ^14^C release with minimum weight loss. In the present work the behaviour of virgin Pile Grade A (PGA) graphite under various thermal oxidation conditions is studied to investigate the potential use of this technique for future examination of the distribution of ^14^C in irradiated material, not as thermal treatment method but as a characterisation method.

### Graphite Oxidation

Unlike fuels such as coal and charcoal, graphite itself is not readily combustible due to the lack of volatile hydrocarbons or oxygen [13]. However, when exposed to various gaseous environments at suitable elevated temperatures [14] this polycrystalline material will undergo thermal oxidation. This behaviour has been widely studied using various different oxidising gas species, different types of graphite and different temperatures. The effect of impurities, which may act as catalysts to influence oxidation rates and onset temperatures has also been investigated. The reactive gas species of greatest previous interest have been oxygen (O_2_), carbon dioxide (CO_2_) and water vapour (H_2_O) as well as the primary gaseous products of the arising graphite oxidation reactions: carbon monoxide (CO) and hydrogen (H_2_) [15–17]. Graphite will even react in air, where the dominant oxidising species is found to be oxygen compared to water vapour or carbon dioxide [16, 18], Table 2. For all reactant gases, there are several physical and chemical processes that occur during the reaction with graphite [17, 19]. First, the gases diffuse from the flow region to the external surface of the graphite and subsequently diffuse into the graphite and adsorb onto active sites on the internal pore surface. Secondly, the adsorbed gas molecules chemically bind with the surface carbon atoms to form CO or CO_2_ gas. Finally, the generated gaseous products rapidly desorb from the surface and diffuse out of the graphite. The latter two processes (adsorption and desorption) are believed to govern the oxidation rate. Also, if the gaseous reaction products are allowed to accumulate, there becomes an increasing competition for adsorption sites on the graphite which reduces the reaction rates for oxidation. The adsorption process on graphite is believed to be driven by chemisorption rather than relatively weak physiosorption, due to the stronger interactions available from the structure (not weak Van der Waal’s forces) [20]. This is discussed in detail by El-Genk *et al.* [21].

**Table 2:** Relative reaction rates between different oxidising gases and graphite at 0.1 atm and 800°C [16].

Reaction	Relative rate
Oxygen gas plus carbon	10000
Water vapour plus carbon	3
Carbon dioxide gas plus carbon	1
Hydrogen gas plus carbon	0.003

The reaction between graphite and air is believed to have three distinct modes or regimes of oxidation dependent on temperature [14, 15, 17, 22–24]. These being referred to as i) the chemical rate, ii) in-pore diffusion and iii) boundary layer regimes for increasing temperatures. However, there are variations in the transition temperatures between these regimes reported from different studies, Table 3, although previous studies have been focussed on isotropic or near-isotropic graphite (IG and NBG grades) rather than anisotropic graphite such as PGA. This is likely due to the transition between the regimes not being fixed but being a gradual shift with suggestions that the transition temperatures are affected by the microstructure, impurity content and density of the graphite [25] as well as the reactive gas flow rate and conditions [19].

**Table 3:** Temperatures of the three different regimes of graphite oxidation sourced from different studies.

Study	Regime 1 Temperature °C	Regime 2 Temperature °C	Regime 3 Temperature °C	Graphite Studied
[16]	Not stated	<750	>750	IG-110
[18]	<500	500–900	>900	IG-11
[14]	450–600	600–900	>900	NBG-17, NBG-25, IG-110 and IG-430
[15]	400–600	600–900	>900	Literature only, no experimental research
[20]	<700–825	825–1371	>1371	Literature only, no experimental research
[23]	400–600	600–800	>800	IG-11
[26]	<500	500 -900	>900	NBG-10 and NBG-18
[27]	<650	650–750	Not stated	PGX, NBG-10 and AG-13-01

Even though there are disparities in the reported transition temperatures of the regimes there are agreements in what the three regimes are. The first regime, referred to as the **chemical rate regime**, occurs at lower temperatures and exhibits a slow reaction rate between the oxidising species and the graphite, thereby allowing transport of the gaseous species deep into the pore network [17]. This allows the concentration of the oxidising species and hence the oxidation to be evenly distributed throughout the bulk of the material [23]. This uniform reaction increases the volume porosity and size of the volume pores without changing the external geometry of the graphite [21]. During this regime the reaction rate will increase rapidly with an increase in temperature due to the reaction being totally determined by the intrinsic reactivity of the graphite [15]. In addition, due to the free availability of oxygen, the product is almost entirely carbon dioxide [28].

The third regime, referred to as the **boundary-layer controlled regime**, occurs at high temperatures (>900°C) and is attributed to the oxidation rate at the surface being so high that oxidation is almost entirely limited to the external surface of the graphite [14, 15] and diffusion into the porous network effectively ceases [21, 23]. This leads to the external surface being preferentially attacked and the geometry changing [17, 18] but with no significant effect on the internal pore structure [21, 23]. In this regime the oxidation rate of graphite only changes slowly with increasing temperature due to the limited availability of the oxidising species [17].

In between these two regimes there is the **in-pore diffusion regime**, whereby the oxidation rate is limited by both chemical rate and diffusion rate [18, 23]. Due to the counter current diffusion of reaction products out of the graphite the oxidants cannot readily penetrate deep into the pores [21]. Therefore the concentration of the oxidising gas and the consumption of graphite decreases with increased depth [17]. Also as the temperature increases an increasing proportion of the gaseous off-product is carbon monoxide due to the increasing reaction rate and oxygen deficiency. Since two molecules of carbon monoxide are formed for every split oxygen molecule, there is an increased net flow of off-gas out of the pores, further inhibiting the diffusion of oxidants deep into the pore volume [28, 29]. This leads to the increase of the oxidation rate with rising temperature being reduced compared to regime 1, but due to the lower activation energy the total oxidation rate is higher [21].

As attested to by the wealth of available literature, the oxidation behaviour of graphite has been well studied with virgin nuclear material grade graphite as a popular material. However, it would be inappropriate to use these results alone to predict the thermal behaviour of reactor-aged irradiated PGA graphite. There are two reasons for this; firstly the thermal oxidation of PGA graphite has not been studied extensively in the past, with large amounts of the research performed on different grades of graphite, mainly the IG (110 and 43) and NBG (10, 17 and 25) grades. There have been studies using X-ray tomography to investigate thermally oxidised PGA graphite, however these have been focussed on using thermal oxidation to replicate radiolytic oxidation in order to determine microstructural evolutions [30], density variations [31] or to automate porosity identification and segmentation [32] in PGA graphite. Berre *et al.* [33, 34] also used thermal oxidation and X-ray tomography to study gilsocarbon graphite, used in later AGR reactors, but again this was to replicate radiolytic oxidation to develop models for failure analysis. The work presented here varies from these as the aim is to understand the thermal oxidation behaviour of the graphite to develop a method for studying radioisotope distribution in irradiated graphite. This investigation into the specific oxidation behaviour of PGA graphite is crucial as different grades of nuclear graphite have different precursor materials and different manufacturing processes there are significant differences in the microstructure and impurity levels of the graphite [35]. Since the oxidation behaviour of graphite is significantly influenced by microstructure [36], a thorough understanding of the oxidation behaviour of the specific grade graphite of interest is necessary. Secondly, there have been no reported oxidation studies of irradiated PGA graphite which may be influenced by many factors, one of which being the impurity content. This may be significant as one major area of previous research focus has been on the influence of impurities in graphite on controlling its rate of oxidation. Impurities are intrinsically present in the graphite due to manufacturing processes with the nature and concentration of these impurities very much dependent on the type of graphite, there are also large variations in the impurity content of different types of graphite and between different manufacturing batches of the same type of graphite. As the irradiated ex-service graphite is not likely to be from the same batch as the virgin material examined here, there may be different impurity concentrations present at start of life and then throughout lifetime in the reactor, as contamination and transmutation will lead to further variations in impurity concentrations. The most notable effect is that impurities can exert catalytic behaviour on oxidation whereby they reduce the activation energy and increase the rate of oxidation. Most prominently metallic impurities are known to act as catalysts in the gasification of graphite. There are two proposed mechanisms for the catalytic behaviour of these metal and metal-oxides on graphite oxidation; either the impurities interact with the surface of the graphite and the available *pi*-electrons, altering the bonding behaviour [37–39] or carbon is oxidised by metal oxide in an oxidation-reduction cycle [37, 40].

There have been many studies [38, 39, 41, 42] on this phenomenon with one of the most detailed being by Heintz and Parker [37] who studied the oxidation of type UCP-1-200 (Ultra Carbon Corporation) graphite in air in the presence of 0.1 mole per cent of 42 different transition metal and inner-transition (f-block) metal-oxide impurities. The authors investigated both the activation energy and reaction rate at 600°C and 700°C and determined that metals having 5 or 10 electrons in their (n-1)d orbital will be active catalysts e.g. chromium, manganese, copper and zinc amongst others. The catalytic effect of selected f-block compounds on the oxidation of graphite in air was also researched by Sampath *et al.* [38, 39]. It was shown that all of the compounds studied had a catalytic activity that reduced the temperature at which oxidation was observed from 640°C in the unadulterated parent graphite to as low as 410°C when combined with uranium- and thorium-containing compounds.

This present work aims to understand the thermal oxidation behaviour of virgin PGA graphite to enable a method for a thermal oxidation process to be confidently developed, that can be used in future work on irradiated PGA graphite samples.

## Materials and Methods

### Sample Preparation

**Fig 1:**
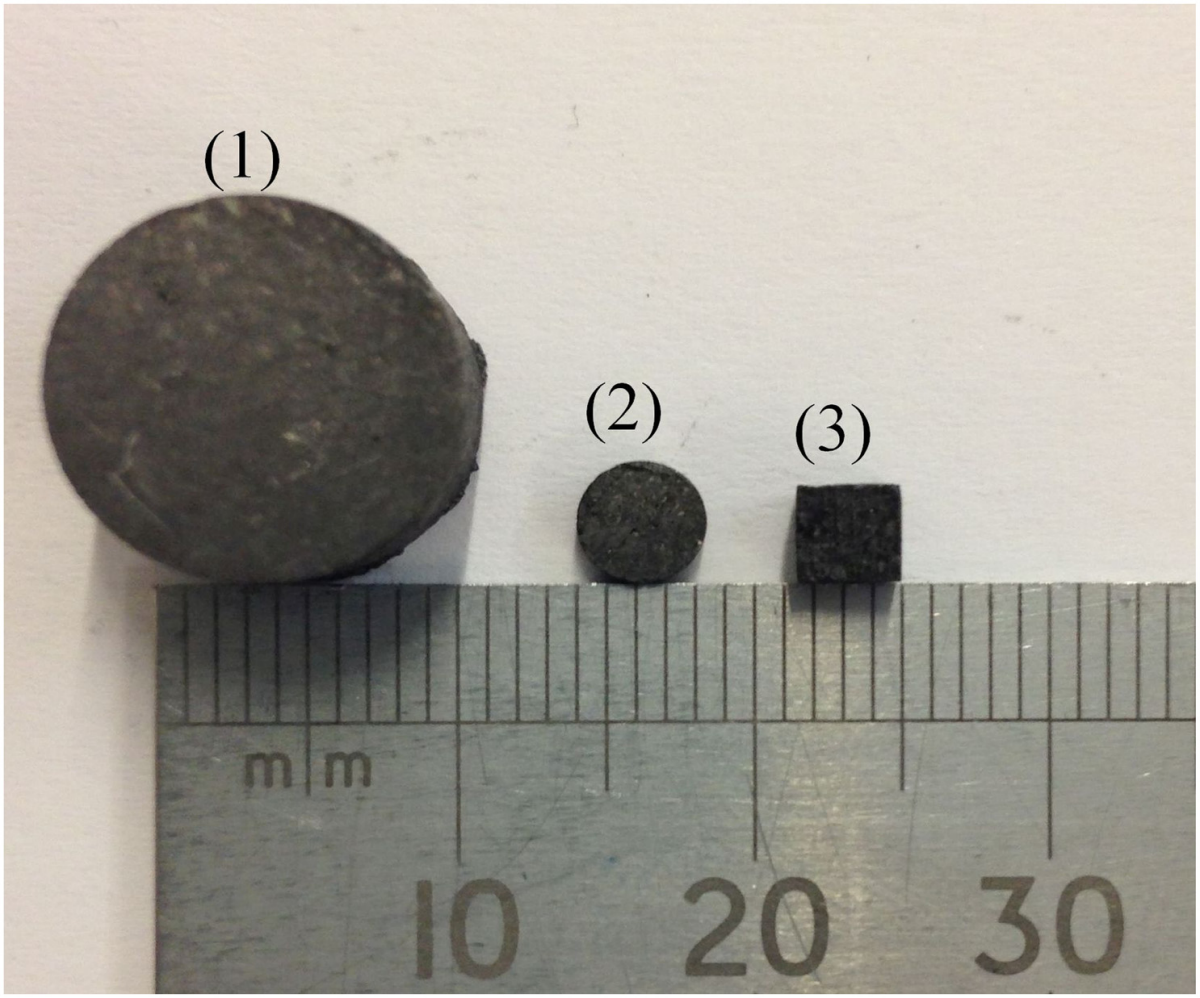
Virgin PGA machined for analysis; (1) 12mm x 7mm discs, (2) 4mm x 2mm discs and (3) 4mm x 4mm x 4mm cubes.

Virgin PGA graphite was supplied by Magnox Limited as a surplus material from Wylfa Power reactor, Wales. This was prepared for analysis in three ways Fig 1, first it was machined into small cubes with the use of a bandsaw to have approximate dimensions of 4 mm x 4 mm x 4 mm. Secondly, coring drills were used to remove cores with a 4 mm diameter, these were then machined into 2 mm high discs with the use of a South Bay Technology Inc. Model 650 low speed diamond cutting wheel with deionised water used as coolant. Both sets of samples were machined to fit into aluminium oxide analysis crucibles with a 70 μL capacity and subsequent examination using scanning electron microscopy (SEM). Thirdly, larger discs, 12 mm diameter and 7 mm high, which were more suitable for X-ray tomography (XRT) analysis, were prepared in a similar manner to the smaller discs. The surfaces were not polished or prepared in anyway after this and so the cubes had a rough topography whereas the discs had a flat surface and there was no specific directionality.

### Thermogravimetric Analysis

**Fig 2:**
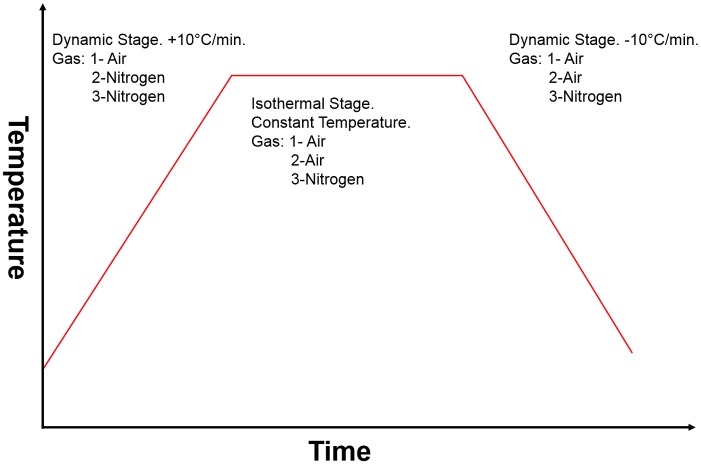
Experimental methods for different gas mixtures.

A Mettler Toledo TGA/DSC1 large furnace thermogravimetric analyser (TGA) was utilised to study the oxidation behaviour of this graphite in the temperature range 600°C–1150°C. Samples were run at various different heating regimes in different gas environments, Fig 2

Each run consisted of a dynamic heating stage followed by an isothermal stage and then a dynamic cooling stage. Of main interest was the stage where the sample was held at a constant temperature for a set period of time as the arising data allowed for a rate of oxidation to be calculated. The oxidation rates were calculated during the 3600 second isothermal stage and calculated as a function of percentage mass loss per second because variations in initial masses between samples invalidated the use of mass units alone. The initial masses of the samples were measured using the XP5U, 5.0 g limit/ 0.1 *μ*g resolution, balance (Mettler Toledo) incorporated in the TGA instrument. Samples were then heated at 10°C/min starting from room temperature (approximately 25°C) up to a final temperature of 600°C with air being introduced at 25 mL/min as a reactive gas. This was held for a 3600 second period with a continued flow of 25 mL/min of reactive gas. The samples were then cooled at -10°C/min until they returned to room temperature. This was repeated for a fresh graphite sample four times each for the cubes and the discs, giving a total of 8 analyses for each temperature and gas mixture. A new set of 8 samples was then examined at a final temperature of 650°C, the final temperature was then increased in 50°C intervals for a fresh set of samples up to a maximum of 1050°C [Experiment 1]. A control experiment following the same sequence as detailed above was run on the cuboidal samples only using dry nitrogen instead of air (flow rate 25 mL/min) for the entirety of the experiment [Experiment 3]. Additionally, an intermediate experiment [Experiment 2] was performed by heating the graphite sample in a flow of dry nitrogen (flow rate 25 mL/min) and then switching the reactive gas to air once the final temperature had been achieved (flow rate 25 mL/min). Two larger disc samples were oxidised using the TGA instrument in several experimental runs in a flow of 25 mL/min air at 650°C and 900°C, these samples were weighed before and after using the balance described above. The duration of each run for each sample varied, due to the different oxidation rates, with each sample oxidised until it reached an approximate total weight loss of 18%, determined by recording the mass. XRT analysis was performed pre and post each oxidation run to generate time resolved data.

### Scanning Electron Microscopy

A Helios NanoLab 600i combined SEM/FIB system (FEI, Oregon USA) was used to obtain scanning electron micrographs. Electron micrographs were acquired using an accelerating voltage of 15 kV and an electron beam current of 0.17 nA.

### X-Ray Tomography

XRT analysis was performed by the National Composites Centre (NCC), Bristol, UK. A Nikon XTH225ST Computerised Tomography X-ray Scanner (Nikon Corporation, Japan) with a maximum focal spot size of 3 *μ*m was used to examine bulk microstructure of pre and post oxidation samples. An energy of 60 kV and current of 160 *μ*A were used throughout with an exposure time of 250 ms for 3142 projections. Avizo 3D visualisation software was used for visualising the arising tomography data.

## Results

### Nitrogen control experiment

**Fig 3:**
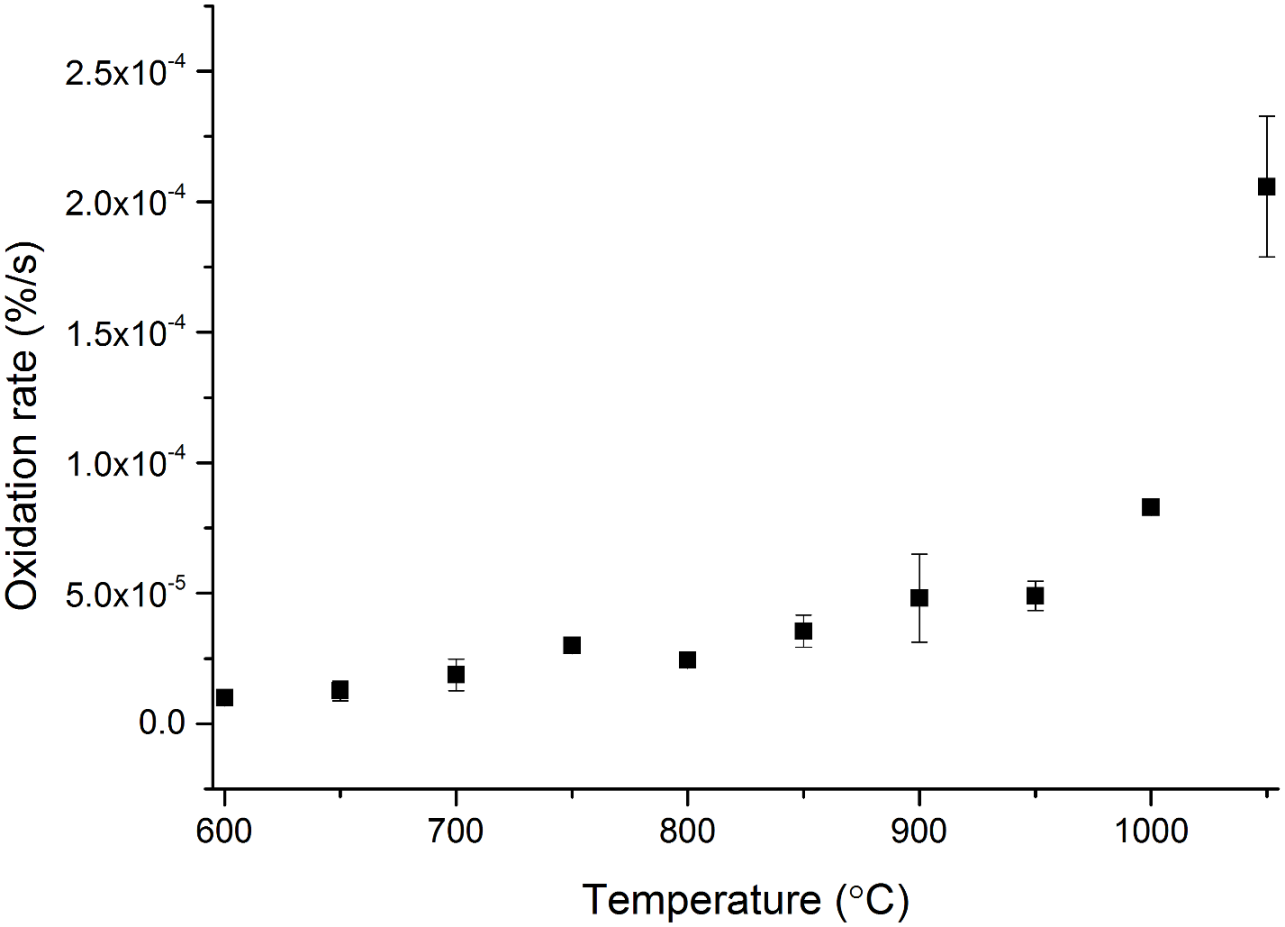
Oxidation rates of virgin PGA graphite during the nitrogen control experiment (experiment 3). Error bars are from calculated standard error of the mean.

The control experiment in nitrogen was performed solely on small cuboidal samples over the temperature range 600°C–1050°C, Fig 3. These showed that there was no significant oxidation of the graphite up to 1050°C, <0.00025 %/s, with this insignificant amount of oxidation ascribed to ingress of trace amounts of air from the environment as the experimental system is not completely sealed. This conforms with the previous literature that the reactions causing weight loss in the graphite must involve an oxidising species such as oxygen or carbon dioxide. However, this conclusion is only valid at the temperatures studied as the reaction between nitrogen corroborated graphite has been observed, but only at temperatures in excess of 1500°C [43].

### Oxidation rates in air

**Fig 4:**
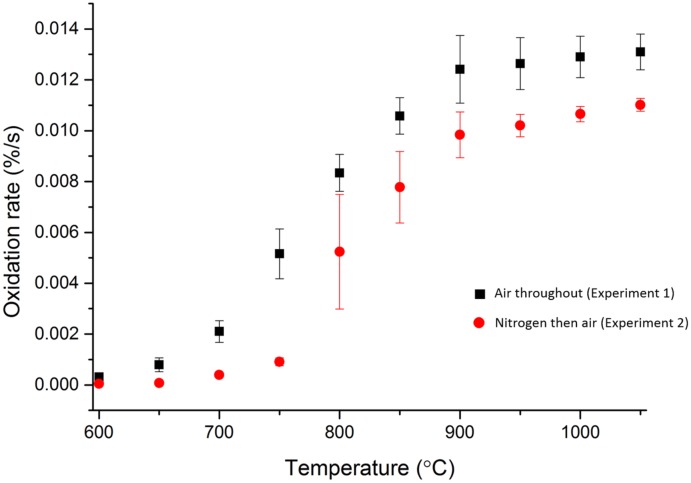
Oxidation rates of cuboidal virgin PGA graphite samples in air, black squares, and in air following heating in nitrogen, red circles. Error bars are from calculated standard error of the mean.

**Fig 5:**
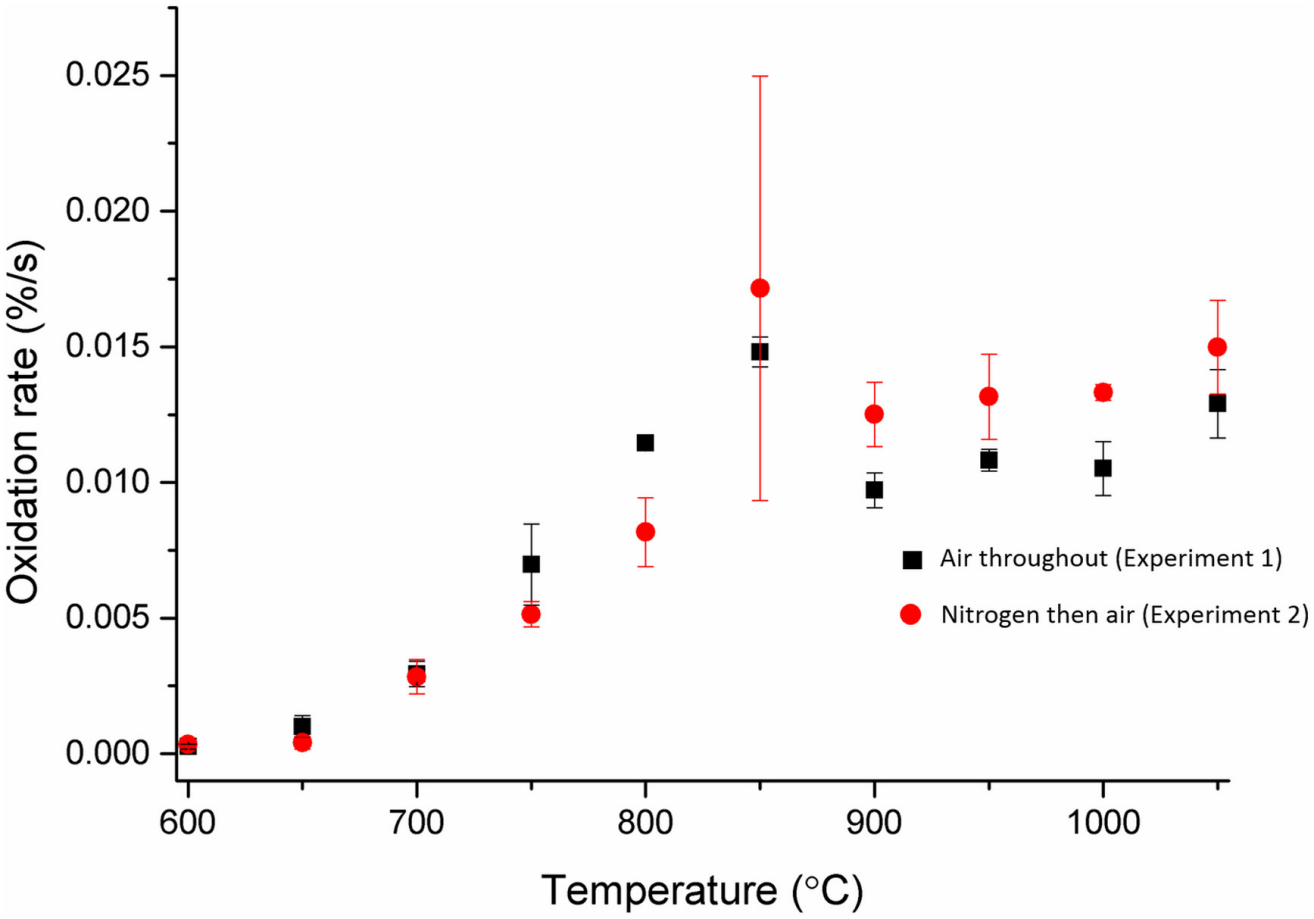
Oxidation rates of small disc virgin PGA graphite samples in air, black squares, and in air following heating in nitrogen, red circles. Error bars are from calculated standard error of the mean.

Results from both cuboidal and disc samples, Fig 4 and Fig 5 respectively, for the two experiments where air was used as the reactive gas followed a similar pattern which was distinctively different from the control experiment. At low temperatures, ∼600°C, very little oxidation occurred, as there would be insufficient energy to overcome the activation energy required for oxidation reactions to occur. However, with increasing temperature there was an approximately linear increase in the oxidation rate until ∼900°C, where a plateau was recorded with very little increase in the oxidation rate as a function of increasing temperature. The pattern observed strongly conforms with the observations described in the literature [23], where the initial oxidation rate is low at lower temperatures due to the reaction being in the chemical rate regime. The oxidation rate then increases with temperature as the reaction moves into the in-pore diffusion regime until at higher temperatures there is an approximate plateau in the oxidation rate, marking the transition into the boundary layer controlled regime. The plateau is due to oxidation rate in the boundary layer regime being controlled by the availability of the oxidising species, meaning changes in temperature had very little effect on the overall oxidation rate.

A notable difference was observed between cuboidal samples heated in air for the duration of the experiment, experiment 1, compared to those heated in a flow of nitrogen and subsequently exposed to air, experiment 2. The oxidation rates of samples heated in nitrogen were lower than those samples heated in air which is believed to be due to nitrogen interfering with the reaction with oxygen. Molecules of nitrogen will occupy the total pore volume and also adsorb onto the surface of the graphite. When a flow of air is introduced, nitrogen will diffuse out at approximately the same rate as oxygen from the air diffuses in, due to the similarity in masses as described by Graham’s law [44]. Therefore oxygen would not be instantly available within the graphite for adsorption and subsequent reaction and this would affect the overall reaction rate, which was measured over a relatively short duration. Once the oxygen had diffused into the graphite, oxygen molecules could form surface complexes with the carbon molecules of graphite [45], but only if sorption sites were available. For graphite that was purged and heated in dry nitrogen, the pore surfaces will have been covered by adsorbed nitrogen (or other impurity gases) which would have had to desorb prior to adsorption of oxygen. Again this lead to oxygen not being instantly available for reaction and thus would affect the observed rate of oxidation for the short duration experiment, as seen by the experiment with the cuboidal samples, Fig 4. This observation was seen in the examination of disc samples, Fig 5, up to 900°C. However, at higher temperatures this conclusion cannot be made as there are greater uncertainties in the results and it is likely any effect of nitrogen interference is lessened due to the reduced surface area and thickness of the disc samples, meaning at higher temperatures the reaction occurs rapidly for both experiments.

### Post oxidation analysis

**Fig 6:**
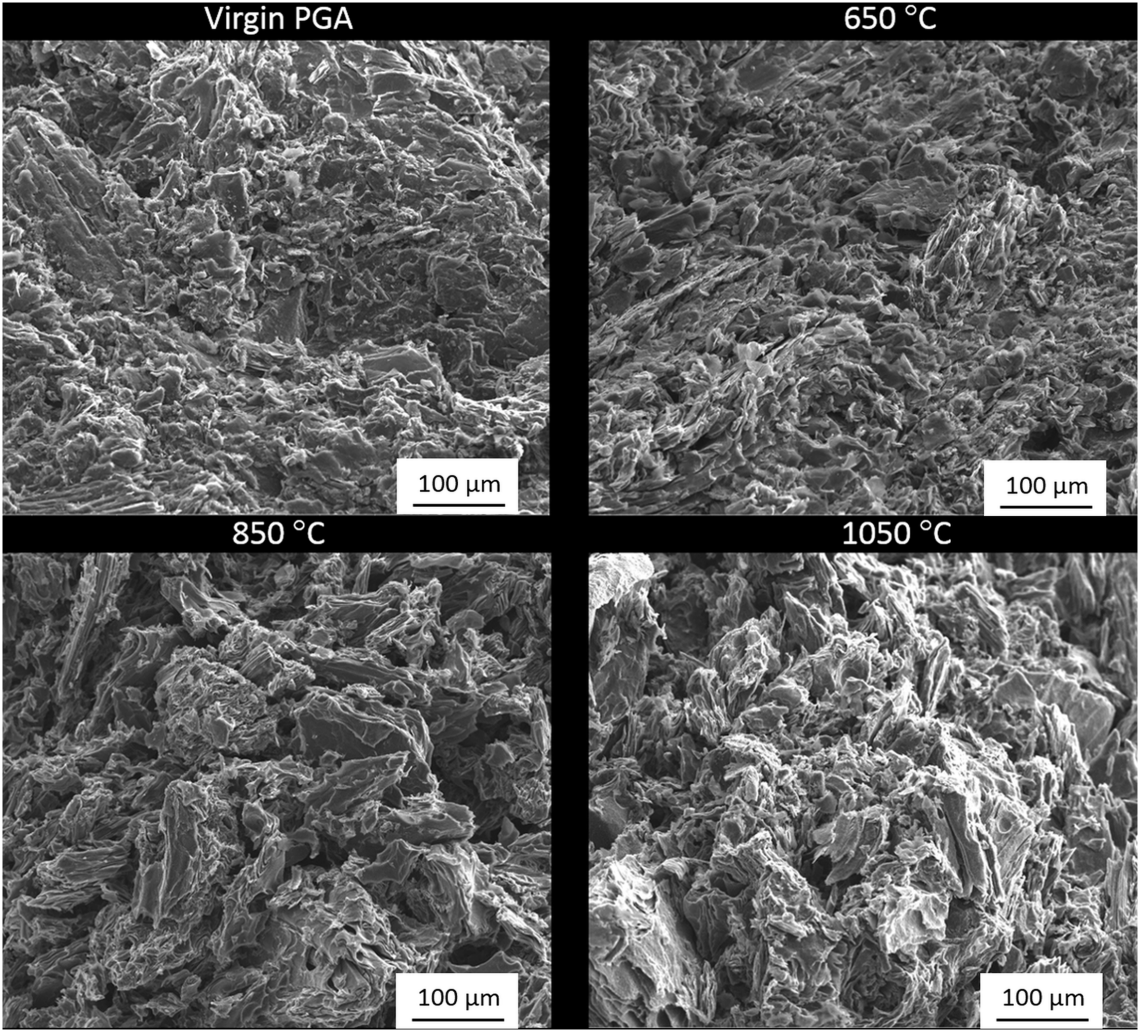
Electron micrographs from PGA graphite oxidised in air at different temperatures along with virgin material for comparison.

Post oxidation analysis was performed through a combination of SEM and Raman spectroscopy. Electron micrographs were collected from samples oxidised in air, Fig 6, at 650°C, 850°C and 1050°C along with a virgin PGA sample for comparison. It was observed that with increasing oxidation temperature there was an associated increased loss of material from the outer geometrical surface of the graphite. This is in good agreement with the TGA results presented and the oxidation models discussed in previous literature. When oxidation is in the boundary-layer regime it leads to material loss almost entirely from the external surface due to the diffusion of oxidising species into the pore network being limited. Samples oxidised at 1050°C are characteristic of this phenomenon with the external surface being vastly different from both the virgin material and the samples oxidised at lower temperatures. Even though the internal pore structure is not visible it is believed that oxidation occurred predominantly in the boundary-layer controlled regime as the sample lost approximately 92% of its original weight with the sample geometry changing significantly, indicating the oxidation was from the external surface with material being lost progressively following a shrinking envelope regime. At lower temperatures, 650°C, the chemical rate regime is anticipated as being the predominant method of oxidation. Samples oxidised at these low temperatures showed very little change in surface topography and size compared to virgin material however they still lost 6% of their initial mass suggesting that this weight loss had occurred evenly throughout the bulk of the material including the pore structure. Control by both boundary layer diffusion and chemical rate occurred at intermediate temperatures with samples oxidised at 850°C exhibiting characteristics of both regimes. With a total recorded weight loss at approximately 57%, the external surface of these samples displayed signs of oxidation. However, the sample geometry did not show such significant morphological change at higher temperatures suggesting that oxidation and associated weight loss occurred both on the surface and within the pore network, although not as deep into the pore network as at lower temperatures.

**Fig 7:**
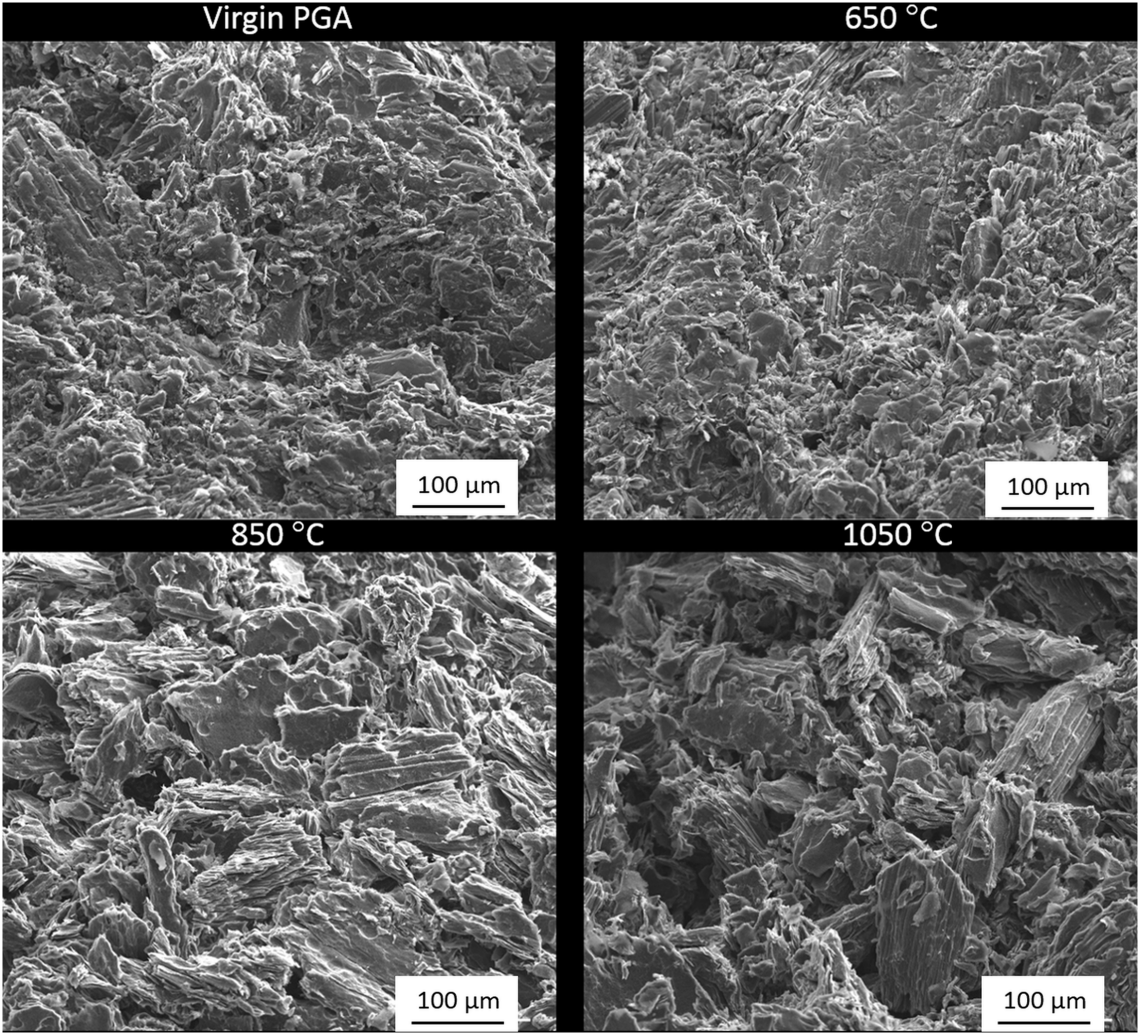
Electron micrographs from PGA graphite oxidised in air following flushing with nitrogen at different temperatures along with virgin material for comparison.

Graphite samples that were heated in nitrogen before being exposed to air, Fig 7, showed very similar characteristics to those exposed to air for the entirety of the experiment. As oxygen is the predominant oxidising species this is not unexpected because the reaction occurs in the same regimes as discussed above. However they have lower weight losses (1%, 23% and 50% for 650°C, 850°C and 1050°C respectively) compared to graphite oxidised in air (6%, 57% and 92%). Oxidation rates were calculated to be lower for graphite under these experimental conditions and, since the experiments were run for the same duration, the total weight loss was lower for these experiments.

The material loss observed during oxidation does not affect the graphite uniformly, especially at higher temperatures. Subsequent to oxidation at high temperatures the remaining material has the appearance of filler particles with the impregnant and pitch binder removed, especially noticeable at 1050°C. This has been previously described in the literature and it has been suggested that the impregnant is more reactive than the binder which in turn is more reactive than the filler [27, 46–48]. This is thought to be due to the the pitch binder and impregnant being more disordered and chemically reactive than the calcined filler particles [27], and therefore has a lower activation energy [46]. This preferential oxidation of the pitch binder is only thought to occur at higher temperatures with Contescu *et al.* [27] stating that at temperatures below 650°C the oxidation rate is practically constant, however in excess of this there is a distinct peak in oxidation rate that is due to preferential oxidation of material.

### X-Ray tomography

**Fig 8:**
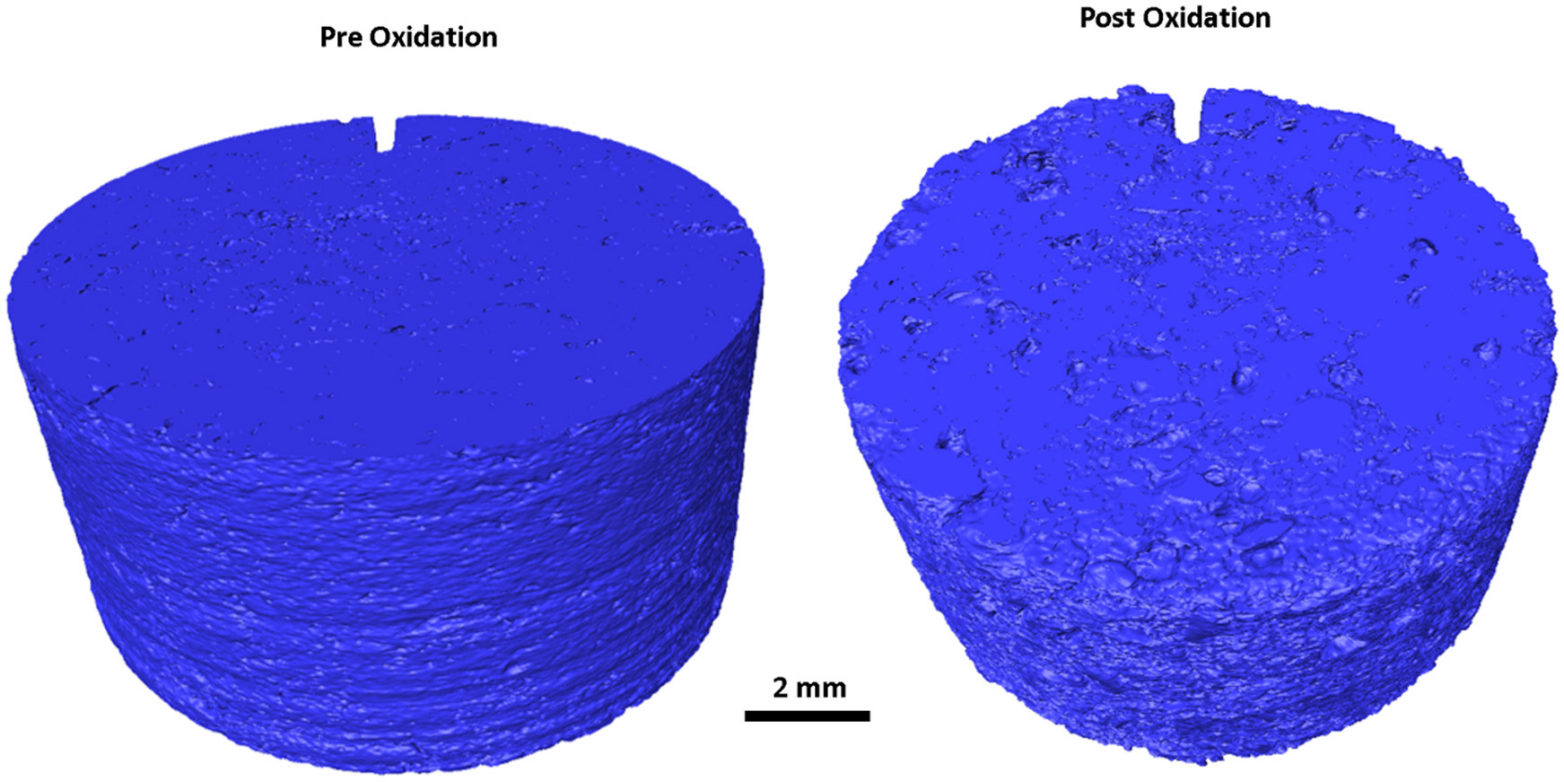
Avizo reconstruction from XRT of PGA graphite prior and post oxidation at 650°C.

**Fig 9:**
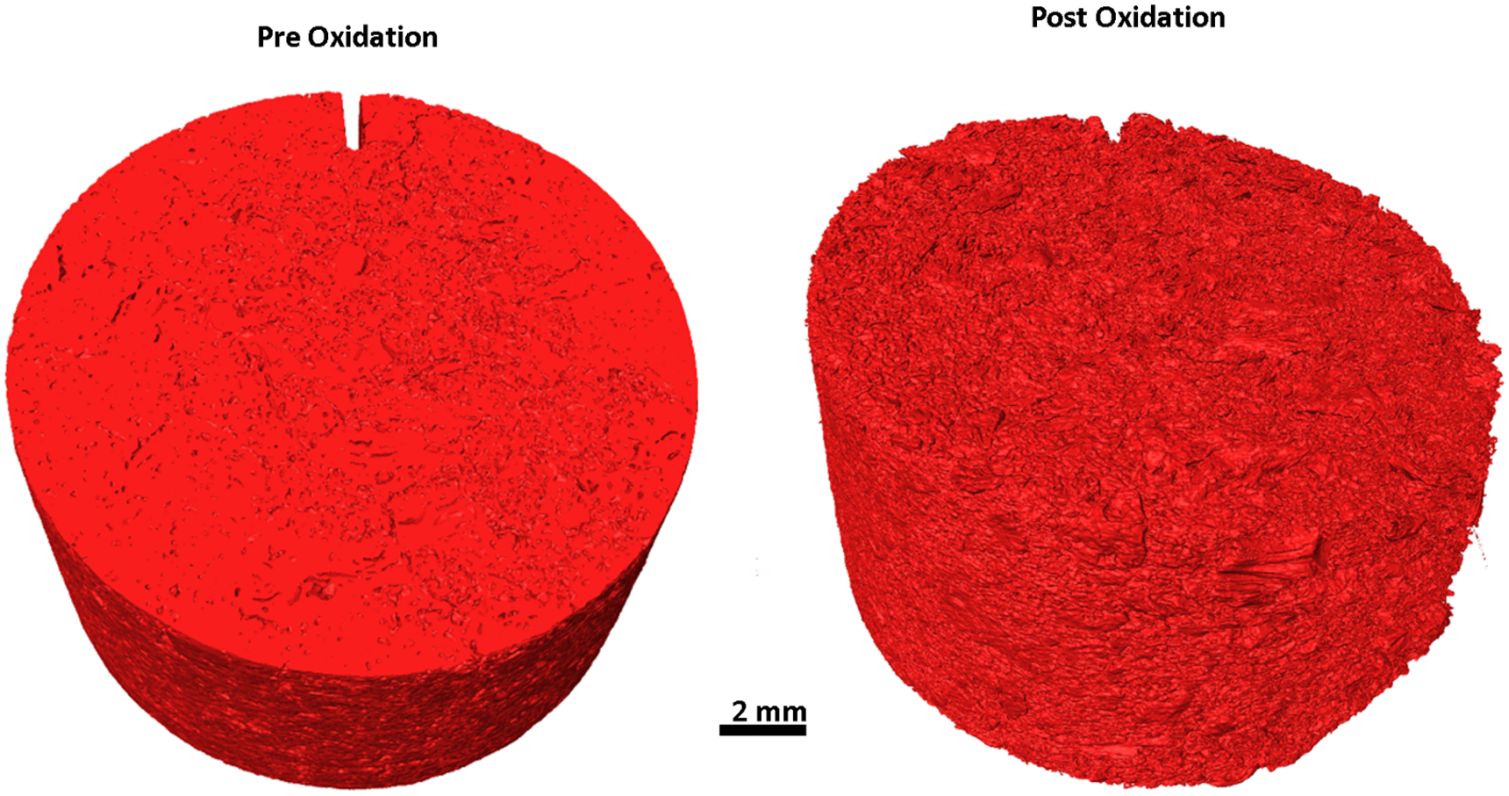
Avizo reconstruction from XRT of PGA graphite prior and post oxidation at 900°C.

**Fig 10:**
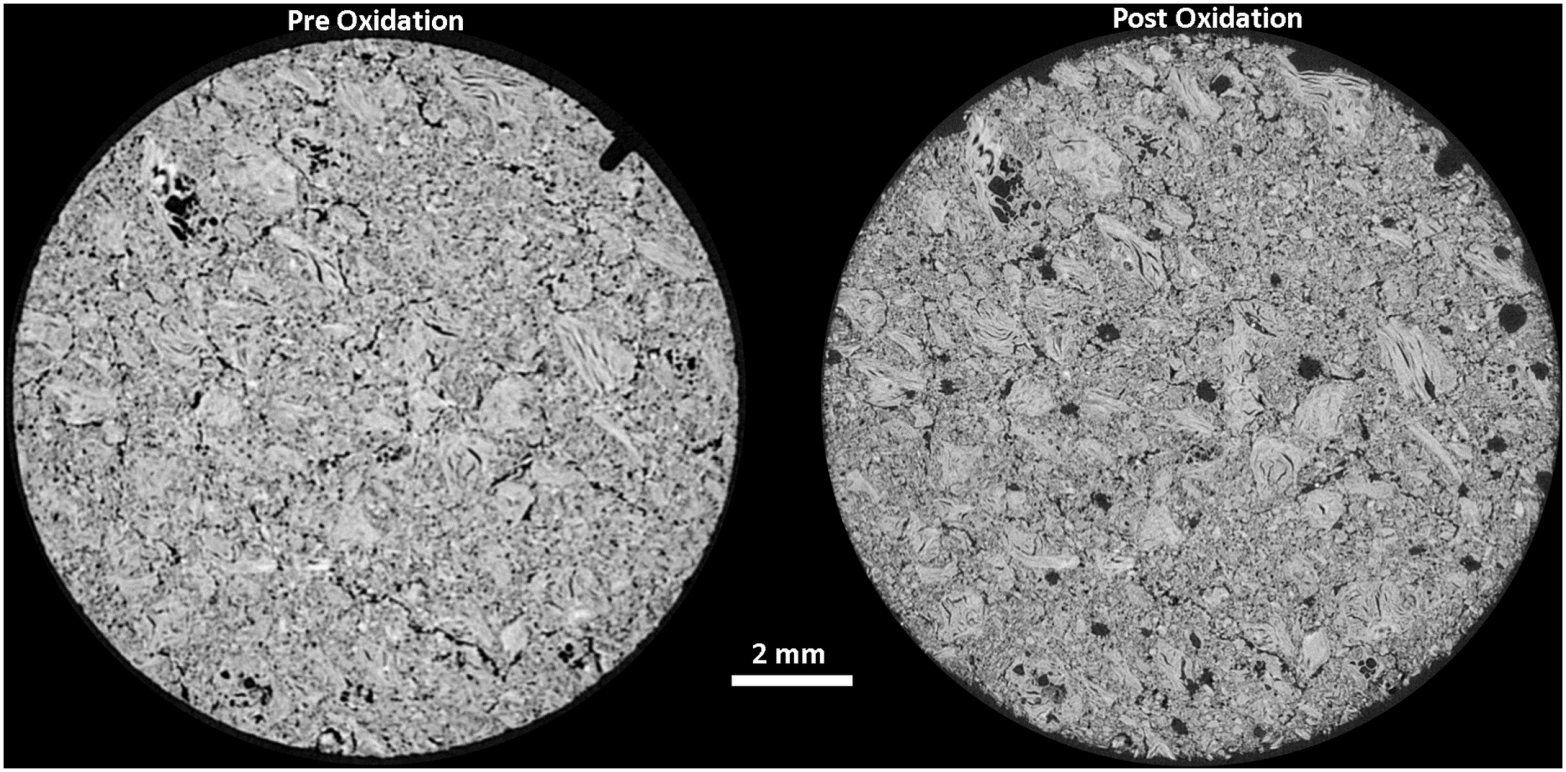
X-ray tomograms from PGA graphite pre and post oxidation at 650°C in air.

**Fig 11:**
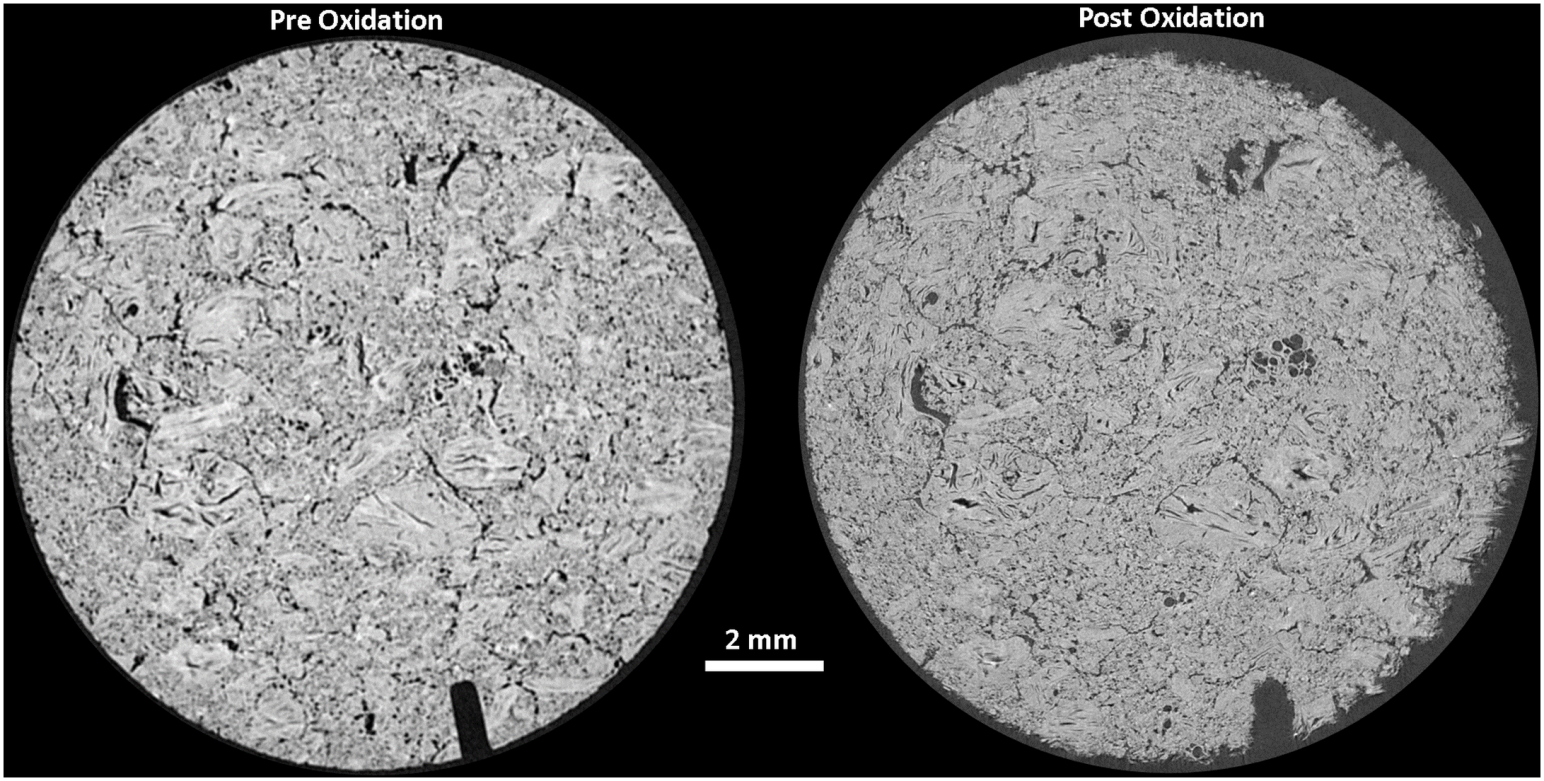
X-ray tomograms from PGA graphite pre and post oxidation at 900°C in air.

Larger disc samples were examined using XRT both before and after oxidation at 650 and 900°C, with the oxidation performed in a flow of 25 mL/min air up to a maximum weight loss of approximately 18%, determined by recording the mass. The total duration of oxidation varied due to the differing oxidations rates with temperature, discussed above, with the sample oxidised at 650°C taking approximately 17 hours to reach 18% weight loss whereas the sample oxidised at 900°C only needed 2 hours to reach 17.9% weight loss. Prior to any oxidation XRT was performed to establish a baseline structure of the material for subsequent comparison, Fig 8 and Fig 9 for the samples oxidised at 650 and 900°C respectively. Both of these show the expected structure of PGA graphite, with a collection of largely interconnected pores dispersed in a solid material. The Avizo reconstructions do not show the individual components such as filler particles or pitch binder due to the similarities in attenuation causing difficulty in determining thresholds in the data processing. However, the tomograms collected from virgin PGA prior to oxidation, Fig 10 and Fig 11, clearly show the presence of large filler particles dispersed in a matrix of flour, binder and pores. At a lower temperature (650°C) there was very little change in the outer geometrical surface size or topography, Fig 8, with only a slight increase in surface roughness visible. At 18% weight loss it is expected that there would be significant removal of material, which is not seen on the outer surface. Therefore it is postulated that the oxidation and associated material loss is occurring within the graphite, this can be seen in the tomograms, Fig 10, which show the appearance of pockets of pores forming within the graphite that were not present in the unoxidised material. Conversely, at higher temperatures (900°C), Fig 9, it is evident that significant oxidation has occurred primarily at the outer surface with quite substantial changes in sample size and appearance. The oxidation of graphite is not uniform with the majority occurring on one side of the sample, this is believed to be due to the TGA furnace having a flow system for the injection of reactive gases. This will lead to oxidising species reacting preferentially on the surface closest to entry, giving rise to the appearance seen in the reconstructions and also in the tomograms, Fig 11.

## Discussion

The oxidation of PGA graphite over a range of temperatures from 600–1050°C was investigated with the use of TGA and SEM along with XRT to better understand the material behaviour and to determine if thermal treatment could form the basis of a technique to examine the concentration and distribution of the radioisotope ^14^C in irradiated graphite retrieved from Magnox nuclear power stations. The oxidation of PGA graphite must involve an oxidising species (O_2_, H_2_O, CO_2_) at these temperatures and will not proceed in an environment that does not contain such species, as demonstrated by the nitrogen-only control experiment [43]. When reacted with air the predominant oxidising species is considered to be oxygen [16]. The results obtained in the present work are in good agreement with results from other authors [14, 16–18, 27] examining different types of graphite. Three regimes of oxidation of graphite are commonly discussed and the TGA results obtained in this work highlight these.

At lower temperatures (<600°C) in the chemical rate controlled regime there is very little oxidation and the rate is slow. At temperatures exceeding approximately 900°C the reaction rate is relatively fast and is boundary layer diffusion controlled, with further increases in temperature having minimal effect on the oxidation rate up to the final temperature studied (1050°C). Luo *et al.* [23] stated that in IG-11 (isostatic molded) graphite there is a further increase in oxidation rate at higher temperatures, however these elevated temperatures were not studied in this work as the main interest is in the lower temperatures. At temperatures between these two (600°C–900°C) is a thermal zone, known as the in-pore diffusion regime, where increasing the temperature has a significant effect on the oxidation rate.

Electron micrographs and X-ray tomography taken of the PGA graphite correlate well with the TGA findings showing that at higher temperatures the weight loss is quite significant and predominantly affects the external geometrical surface causing the sample to reduce in size considerably. At lower temperatures there is very little evidence of oxidation on the surface and no change in sample geometry, however there is still appreciable weight loss of the samples, suggesting that this is occurring evenly throughout the graphite. For the in-pore diffusion regime a combination of the two regimes exist, giving oxidation on the surface and within the pore network.

The area of most interest for future work is the chemical rate controlled regime, operating at temperatures <600°C, due to the relatively slow and uniform oxidation rate. This slow oxidation rate allows diffusion of gases throughout the graphite and deep into the pore network, effectively oxidising the graphite from the exposed outer surface of not only the external surface but also the pore surfaces. This is critical for studying ^14^C concentrations as this radioisotope may not be homogeneously distributed throughout the graphite as ^14^C formed from ^14^N or ^1^O from the coolant gas may be more localised on the pore surface whereas ^14^C from ^13^C and ^14^N found in the graphite lattice will be evenly distributed and also be bonded differently [49]. LaBrier and Dunzik-Gougar [49] studied NBG-25 graphite that had been irradiated in a test reactor at the Idaho National Laboratory. Time-of-Flight Secondary Ion Mass Spectrometry and X-Ray Photoelectron Spectroscopy showed that ^14^C exists predominantly on or near the surface. However, as this was performed on a different type of graphite and on samples irradiated for a short period in a test reactor compared with >30 years exposure in an electricity generating reactor, caution must be taken in comparison of results. Fachinger *et al.* [50] and Smith *et al.* [51] have both studied the possibility of using thermal treatment (selective oxidation) to selectively remove ^14^C from the surface of irradiated graphite and even though this work was aimed at decontamination of graphite prior to disposal it highlights that under suitable conditions ^14^C can be removed and gases collected and subsequently analysed.

In the present work on virgin PGA the approximate temperatures for each previously documented thermal oxidation regime were determined. Care must be taken if these results are assumed to be representative of the expected oxidation behaviour for ex-service irradiated graphite due to it being significantly different to the virgin material in several ways. During the lifetime of the reactor, radiolytic gasification causes significant weight loss with associated pore volume increase and geometrical changes to the graphite [52], this increase in porosity may affect the oxidation rate as it will increase the quantity of oxidising gases that can migrate through the graphite as well as decreasing the reactive surface area since the structure changes from small interconnected pores to larger open pores. Not only does the irradiation by fast neutrons cause gasification, it also leads to radiation damage of the graphite, with associated displacements of carbon atoms from their normal lattice positions, that in turn has some catalytic effect in increasing the oxidation rate by about six times in previously irradiated samples [53]. Although the authors do not provide an explanation for this phenomenon, apart from mentioning that it is not due an increase in surface area, this is an important observation. A similar increase in oxidation rate post-irradiation was observed by Lang *et al.* [54], who suggested annealing of defects in the graphite by irradiation may cause this increase in oxidation rate. Although other authors [13, 15, 55, 56] mention that irradiation may affect oxidation they give no explanation, leaving this phenomenon poorly understood.

The results presented here give an increased understanding of the oxidation behaviour of PGA graphite in air, however caution is required in taking results from this study on unirradiated graphite and applying them to irradiated material as this material is known to have several differences, discussed above, to the virgin material that may affect oxidation rates and temperatures.

## Conclusions

Thermogravimetric analysis, X-ray tomography and scanning electron microscopy were used to study the oxidation behaviour of unirradiated PGA graphite over the temperature range 600–1050°C. Several key conclusions can be drawn:

- PGA graphite will oxidise in air at temperatures in excess of 600°C.
- Results obtained for PGA graphite correlate well with results from other graphite grades and the models described by other authors.
- Oxidation at relatively low temperatures (∼600°C) has the potential to form the basis of an experiment to examine ^14^C distribution in irradiated graphite as it will uniformly oxidise the active surface area of the whole graphite. This will first remove the surface and near surface associated ^14^C before eventually releasing the ^14^C in the graphite lattice.
